# The Effect of a High-Intensity PrO2Fit Inspiratory Muscle Training Intervention on Physiological and Psychological Health in Adults with Bronchiectasis: A Mixed-Methods Study

**DOI:** 10.3390/ijerph18063051

**Published:** 2021-03-16

**Authors:** Jessica L. McCreery, Kelly A. Mackintosh, Rebekah Mills-Bennett, Melitta A. McNarry

**Affiliations:** 1 Applied Sports, Technology, Exercise and Medicine Research Centre, College of Engineering, Swansea University, Swansea SA18EN, UK; jessica.l.mccreery@swansea.ac.uk (J.L.M.); k.mackintosh@swansea.ac.uk (K.A.M.); 2 Physiotherapy Department, Glangwili Hospital, Dogwili Rd Carmarthen, Carmarthen SA312AF, UK; Rebekah.Mills-Bennett@wales.nhs.uk

**Keywords:** bronchiectasis, inspiratory muscle training, chronic disease, respiratory, quality of life, mixed methods

## Abstract

Bronchiectasis is characterised by airflow obstruction and hyperinflation resulting in respiratory muscle weakness, and decreased exercise capacity. Inspiratory muscle training (IMT) is potentially an alternative treatment strategy to enhance respiratory muscle strength and endurance. Therefore, the aim was to investigate the effects of IMT on those with bronchiectasis. Eighteen participants (10 bronchiectasis) took part in an eight-week, three times a week IMT programme at 80% sustained maximal inspiratory pressure (SMIP). Lung function, respiratory muscle strength and endurance, exercise capacity, physical activity and self-determination theory measures were taken. Participants also took part in a semi-structured interview to assess their perceptions and experience of an IMT intervention. After eight weeks of IMT, bronchiectasis and healthy participants exhibited significant increases in MIP (27% vs. 32%, respectively), SMIP (16% vs. 17%, respectively) and inspiratory duration (36% vs. 30%, respectively). Healthy participants exhibited further improvements in peak expiratory flow and maximal oxygen consumption. Bronchiectasis participants reported high levels of perceived competence and motivation, reporting higher adherence and improved physical ability. Eight weeks of IMT increased inspiratory muscle strength and endurance in those with bronchiectasis. IMT also had a positive effect on perceived competency and autonomy, with bronchiectasis participants reporting improved physical ability and motivation, and high adherence.

## 1. Introduction

Bronchiectasis is a chronic pulmonary disease characterised by airflow obstruction due to the destruction of elastic tissue and smooth muscles of the bronchial walls [1,2], arising from a vicious cycle of transmural infection and inflammation [3]. Primary symptoms include cough, excessive secretions, dyspnoea, exercise intolerance and fatigue [4,5]. These symptoms may, at least in part, be attributable to respiratory muscle weakness also reported in those with bronchiectasis [5,6,7,8], which may lead to discord between respiratory muscle load and capacity [6]. Indeed, decreased respiratory muscle strength is associated with less productive coughing and decreased removal of airway secretions [8,9]. Effective strategies to target and resolve respiratory muscle weakness are therefore needed for those with bronchiectasis.

Pulmonary rehabilitation (PR) has been recommended for those with bronchiectasis. However, patient perceptions on the effects of PR are limited [10]. Nonetheless, following improvements in physical and psychological health post-PR, Sinnerton and Gillen [11] also reported enhanced patient confidence and less dependency on medical resources. However, exacerbations, transport difficulties and lack of motivation were highlighted as barriers to participation in, and adherence to, PR. Inspiratory muscle training (IMT), utilising a restricted airflow breathing technique, has often been used as an adjunct to traditional PR programmes in chronic obstructive pulmonary disease (COPD), leading to greater improvements in exercise capacity than PR in isolation [12,13]. However, whether similar benefits are elicited by IMT in those with bronchiectasis is less clear. Specifically, whilst some report improvements in respiratory muscle strength and endurance, exercise capacity and social aspects of quality of life following IMT [6], others have reported beneficial effects to be limited to improvements in inspiratory and expiratory muscle strength [7], or there to be no additional benefits to those associated with traditional PR in those with bronchiectasis [5]. Moreover, home-based IMT in chronic lung diseases has been shown to have a positive impact on activities of daily living, mobility, and breathlessness, with patients becoming more confident in managing their disease [10]. Therefore, investigating patient perceptions and motivations to participate in an intervention, in conjunction with determining associated physiological and psychological effects, is essential when evaluating the overall effectiveness of an intervention. Overall, further work is required to resolve these equivocal findings, which may be related to considerable methodological differences, such as participant adherence to the IMT protocol, which no study has reported, or the intensity, frequency or duration of the IMT used. Indeed, in those with COPD, interval-based, high-intensity IMT has been shown to elicit greater improvements in respiratory muscle function than low- to medium-intensity protocols [14,15].

The long-term nature of bronchiectasis means that patients must cope with the debilitating nature of their disease over the course of their lives [16]. Indeed, patients with bronchiectasis have reported reduced quality of life and increased symptoms of anxiety and depression [17,18]. Feelings of anxiety have been associated with patients’ perceptions of their health and well-being, whereas depression is suggested to be linked to exercise impairment and breathlessness [18]. Therefore, interventions that improve both patient perceptions and exercise capacity, whilst relieving symptoms of breathlessness, are of paramount importance to the psychological health of patients with bronchiectasis.

Therefore, the aim of the current study was to determine the physiological and psychological effect of an eight-week home-based IMT intervention in adults with bronchiectasis. The secondary aim was to evaluate participants’ adherence to the IMT protocol and their experiences and perceptions of this type of pulmonary rehabilitation programme.

## 2. Materials and Methods

Ten clinically stable bronchiectasis patients, diagnosed by clinical history including high-resolution tomography, pulmonary functions tests, cough, shortness of breath and exertional dyspnoea, were recruited from an outpatient clinic in South Wales to take part in this quasi-experimental trial. Eight healthy participants were recruited from university networks and were required to have no pulmonary or respiratory conditions that may impair exercise capacity. Ethical approval was granted by the North Wes—Liverpool Central Research Ethics Committee (reference 16/NW/0764) and written informed consent was obtained from all participants. Participants were required to attend a testing session at baseline and one following the eight-week intervention. For participants who agreed to take part in an eight-week IMT top-up, a third visit was required at week 16.

### 2.1. Inspiratory Muscle Training Intervention

The PrO2Fit device (PrO2 Health Incorporated, Rhode Island, NE, USA) was chosen due to its ability to provide biofeedback and remote adherence monitoring as well as its capacity for greater IMT workloads throughout the full range of inspiration using a decreasing rest period between breaths [19]. Prior to completing the intervention, participants were given a ~30 min familiarisation training session on how to use the device and the inspiratory manoeuvre technique in order to minimise possible learning effects [20]. The PrO2Fit device incorporates a 2 mm leak to avoid glottal closure during maximal inspiration [21].

At the start of every IMT training session, participants were instructed to complete a baseline maximal manoeuvre to determine their sustained maximal inspiratory pressure (SMIP). Participants were then required to train at 80% of their SMIP throughout each inspiratory effort, thereby facilitating an individualised and progressive nature of training [22]. Inspiratory duration was also measured for each breath. Each IMT session involved up to six blocks of six inspirations, with the rest between inspirations in each block progressively decreasing from 60 s to 45, 30, 15, 10, and, finally, 5 s [22]. If participants failed to achieve 80% SMIP, the training session was automatically terminated. Training was performed three times per week, with a least 24 h between sessions, for eight weeks. At the end of the eight weeks, participants were given the option to complete a further eight weeks during which they were asked to complete IMT once a week. All training sessions were uploaded automatically to the cloud allowing remote monitoring.

### 2.2. Anthropometrics

Physical characteristics including body mass (Seca 220; Hamburg, Germany) and stature were measured to the nearest 0.1 kg and 0.01 m, respectively, with body mass index (BMI) subsequently calculated. Waist and hip circumference were measured to the nearest 0.01 m using an anthropometric tape (Seca, Birmingham, UK) at the narrowest point between the base of the ribs and the iliac crest for waist circumference, and the widest point around the hips for hip girth. Waist-to-hip ratio (WHR) was subsequently calculated.

### 2.3. Physiological Measures

Parameters of respiratory function forced expiratory volume in one second (FEV_1_), forced vital capacity (FVC), FEV/FEV_1_ and peak expiratory flow (PEF) were assessed in a sitting position using a portable spirometer (Micro1, MicroRPM, Kent, UK), measured according to European Respiratory Society Task Force guidelines [23]. Respiratory muscle strength was measured using a portable electronic mouth pressure device (Micro Medical MicroRPM, Kent, UK), with maximal inspiratory pressure (MIP) measured at residual volume and maximal expiratory pressure (MEP) determined at total lung capacity. Each participant was required to complete three maximal inhalations and exhalations; differences greater than 10% or 10 cm H_2_O required an additional effort, with the best inspiration and exhalation selected for subsequent analysis. FEV_1_ and FVC were also expressed as the percentage predicted for age, stature and gender [23,24].

If able, participants were asked to complete an incremental ramp test on a cycle ergometer (ViaSpint 150P; ViaSys Healthcare, Germany) to volitional exhaustion. Following a three minute warm-up at 10 W, the resistance was progressively increased at 10 W·min^−1^. Participants were required to maintain a cadence ~60–70 revolutions per minute (rpm) throughout the test, with the test terminated when the cadence decreased by >10 rpm, despite strong verbal encouragement. Subjective ratings of perceived exertion (RPE) were recorded every minute [25]. Subsequently, participants completed a supramaximal validation bout at 110% of peak power output achieved during the initial cycle ergometer test. Breath-by-breath pulmonary gas-exchange data were collected continuously during the incremental and supramaximal exercise tests (MetaMax 3B, Cortex Medical, Leipzig Germany). Participants were required to wear a facemask, breathing through an impeller turbine assemble (Jaeger Triple V, Hoechberg, Germany), with gas volumes and flow rates continuously sampled at 100 Hz.

### 2.4. Psychological Measurements

Employing self-determination theory [26] all participants completed questionnaires to assess treatment self-regulation and perceived competence in relation to IMT, following the initial eight-week intervention. Specifically, the 15-item treatment self-regulation questionnaire was utilised to assess reasons for completing IMT regularly (i.e., “The reason I would undertake IMT is because I feel that I want to take responsibility for my own health”). The treatment self-regulation questionnaire has three subscales: autonomous regulation, controlled regulation and amotivation. Perceived competence was measured using four single-item indicators (i.e., “I feel confident in my ability to undertake IMT regularly”). Both questionnaires were answered using a Likert scale ranging from one (not at all true) to seven (very true) and are validated for use within clinical populations [27].

Finally, participants were invited to complete a face-to-face, semi-structured interview following the eight-week intervention. If participants failed to complete the intervention, they were still invited to take part in an interview to ascertain their perceptions and barriers to completing the intervention. The interviewer (JMC) asked open-ended questions, seeking clarification or elaboration when required. Questions were centred around their experience with IMT, recommendations for future use and more generic opinions of physical activity and exercise. Interviews were audio recorded and transcribed verbatim.

### 2.5. Data Analysis

#### 2.5.1. Cardiopulmonary Exercise Testing and Supramaximal Verification

Oxygen consumption (${\;\dot{V}O}_{2}$) and carbon dioxide production (VCO_2_) were converted to 15 second averages. The highest moving average of Vo_2_ measured over 15 second was taken as the peak value. The gas-exchange threshold (GET) was determined as the ${\;\dot{V}O}_{2}$ at which there was a non-linear increase in carbon dioxide consumption relative to ${\;\dot{V}O}_{2}$_,_ with an increase in minute ventilation (V_E_)/${\;\dot{V}O}_{2}$ without a concomitant increase in V_E_/VCO_2_ [28] Peak ${\;\dot{V}O}_{2}$ was considered maximal if the Vo_2_ peak achieved during the supramaximal verification did not exceed that achieved during the ramp test by ≥9% [29].

#### 2.5.2. Interview Analysis

All interviews were transcribed verbatim by JLM, using a manual method [30], with interview data analysed using an inductive and deductive approach via direct content analysis [31]. Transcripts were coded line by line and then placed in a relevant overarching category. To ensure a detailed account of participants experiences, similar and/or opposing codes were then organised into themes. An independent author (KAM) undertook a cross-examination of the data to challenge the interpretations, ensuring methodological rigour and that findings were logical and to offer alternative interpretations [32,33]. This process was repeated until an acceptable consensus was reached. The triangular consensus procedures afforded credibility and transferability [34].

### 2.6. Statistical Analysis

All data were analysed using IBM SPSS (IMB Corp, Version 25.0. Armonk, New York: IBM Corp) and are presented as the mean ± standard deviation (SD) unless stated otherwise. Statistical significance was accepted as *p* < 0.05. A repeated-measures ANOVA was used to determine the influence of IMT, and how this differed by group. Pearson’s correlation coefficients were used to assess the relationships between Vo_2max_, and lung function. The minimal clinically important difference (MCID) was calculated using distribution-based methods. Specifically, the baseline SD multiplied by 0.5 was used to determine whether IMT had a clinically significant effect on lung function, and peak ${\;\dot{V}O}_{2}$. The change score divided by the baseline SD score was used to calculate effect size, with Cohen’s d (1988) [35] thresholds used for interpretation (small = 0.2, moderate = 0.5 and large = 0.8).

## 3. Results

Fourteen adults with clinically stable bronchiectasis agreed to take part in this study. One patient withdrew due to an acute exacerbation, two withdrew due to the inability to use the device and one was unable to make the retesting visits. Due to the impact of COVID-19, eight healthy participants could not be retested, but they were invited to take part in interviews. Overall, adherence to the IMT protocol was high, with bronchiectasis patients completing significantly more IMT sessions (95%) than the healthy participants (80%) during the first eight weeks.

The healthy participants were younger than those with bronchiectasis who were characterised by a higher BMI at baseline (Table 1). Furthermore, healthy participants exhibited a higher MEP (*p* = 0.05), FEV_1_ (*p* = 0.002), FVC (*p* = 0.002), FVC %predicted (*p* = 0.03) and Vo_2max_ (*p* = 0.002; Table S1).

### 3.1. Effects of Inspiratory Muscle Training

**Figure 1:**
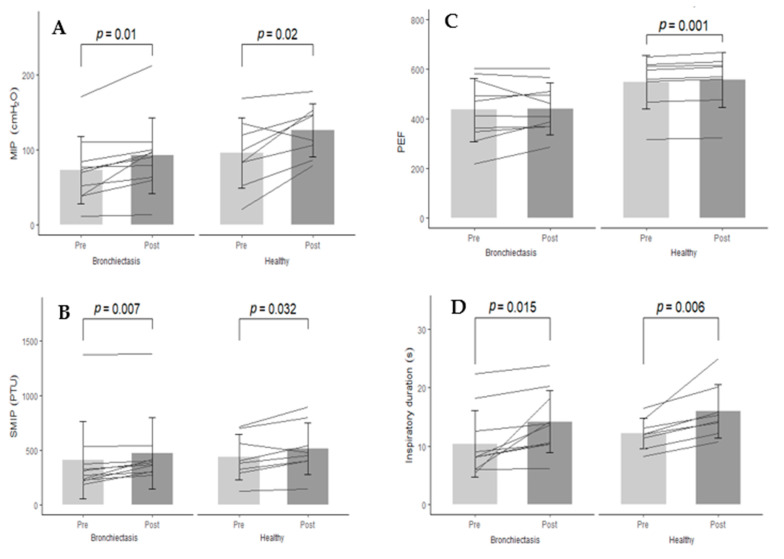
Changes in (**A**) maximal inspiratory pressure (MIP); (**B**) sustained maximal inspiratory pressure (SMIP); (**C**) peak expiratory flow (PEF) and (**D**) inspiratory duration over the eight weeks of an inspiratory muscle training intervention in adults with bronchiectasis and healthy controls. Grey columns represent the mean group response with standard deviation bars. Overlaid lines represent individual responses.

A repeated-measures ANOVA with Greenhouse–Geisser correction showed that eight weeks of IMT was associated with a main effect of time and group for MIP, SMIP and inspiratory duration, with a significant group-by-time interaction for PEF and Vo_2max_ (*p* < 0.05)_._ Specifically, bronchiectasis and healthy participants exhibited significant increases in MIP (27% vs. 32%, respectively), SMIP (16% vs. 17%, respectively) and inspiratory duration (36% vs. 30%, respectively) after eight weeks of IMT. The healthy participants also exhibited further improvements in PEF and Vo_2max_ after eight weeks, which were not evident in the bronchiectasis group (Figure 1; Table S1).

MCID revealed that bronchiectasis patients exhibited small clinically meaningful improvements in MIP, SMIP and HGS (d = 0.4; d = 0.2; d = 0.2, respectively) and a moderate clinically meaningful improvement in inspiratory duration (d = 0.6) following IMT. There were no clinically meaningful improvements in any other parameters (Table 2).

Following the eight-week IMT intervention, MIP significantly correlated with FEV_1_ (r = 0.667; *p* = 0.035) and FVC (r = 0.772; *p* = 0.009), whilst Vo_2max_ showed significant correlations with FEV_1_ (r = 0.693; *p* = 0.039) and FEV_1_/FVC (r = 0.795; *p* = 0.018) in those with bronchiectasis. Contrastingly, in the healthy participants, MIP correlated with MEP (r = 0.821; *p* = 0.012), whilst FEV_1_ was significantly correlated with FVC (r = 0.721; *p* = 0.044).

Compared to healthy participants, those with bronchiectasis exhibited significantly higher levels of autonomy (4.9 ± 1.4 vs. 2.1 ± 0.7; *p* < 0.01) and perceived competence (5.5 ± 1.3 vs. 4.6 ± 1.1; *p* < 0.01), with lower levels of amotivation (1.4 ± 0.6 vs. 4.3 ± 0.4; *p* < 0.01) after eight weeks of IMT (Table 3).

### 3.2. Eight-Week Top-Up Period

Five bronchiectasis and four healthy participants agreed to take part in a further eight-week top-up period. Two bronchiectasis patients subsequently dropped out of the top-up period; one participant was going on vacation and the other had purchased their own PrO2 and wanted to train more frequently. Two healthy participants also dropped out due to being unable to make the final testing visit. Adherence in those that completed the entire top-up period was 100% and 69% for bronchiectasis and healthy participants, respectively.

There were no significant differences in respiratory muscle strength, lung function parameters or exercise capacity between or within groups compared to the values following the initial eight weeks after the eight-week top-up period.

### 3.3. Qualitative Experiences

Key themes derived from the interviews with bronchiectasis and healthy participants are presented below. ‘B’ indicates quotes from bronchiectasis and ‘H’ from healthy participants.

#### 3.3.1. Relationship with Physical Activity and Exercise

##### Positives

Most bronchiectasis participants (80%) and all healthy participants (100%) highlighted a positive relationship with physical activity and exercise, citing experiences of both mental and physical enjoyment:

“I love walking the dogs, because it gets you out into the open air… it is good for you mentally as well I think. It does make you feel better doesn’t it? You feel very virtuous when you’ve done some [physical activity].”

[B10]

“I like the freedom of it… I find it really helpful for my mental health. The knowledge that my body is capable of doing things. I like the sense of achievement.”

[H1]

##### Barriers to Exercise

Symptoms associated with bronchiectasis were highlighted as the main barrier to exercise in this cohort of patients:

“Having this bronchiectasis limits the amount of physical activity and exercise I can do… I look forward to it [physical activity and exercise] mentally, but I am very much aware of my limitations very soon… I wish I could do a lot more walking and I would really enjoy that much more if I could.”

[B3]

With their condition impacting on their activities of daily living:

“I’ve got to take my time on everything, but anything that’s up and down, I mean like the stairs, up is physical, but down is just as hard.”

[B1]

In line with the healthy participants, barriers such as enjoyment, weather, work and time constraints were also highlighted as reasons for being inactive:

“I don’t enjoy it [physical activity], I haven’t got the time to do it. It’s time more than anything [that is a barrier], you come home from work and you’re tired, you don’t want to start doing exercises.”

[B6]

“Time is always the biggest one [barrier to exercise and physical activity]. It always is isn’t it? Time and mental sort of motivation… One of the downsides of my job is that I’m spending a lot of time sitting down. I’ve come from a previously very active job… to then go to a really sedentary job has been really hard and that’s really affected my activity.”

[H5]

##### Motivations to Exercise

Despite these limitations and the barriers to physical activity and exercise, patients understood the importance of being physically active and also highlighted their bronchiectasis as a motivator to be active:

“Since the diagnosis I realise I need to be more active in order to keep my lungs healthy and the sputum moving around... So, the more active you are the better it [bronchiectasis] is. I try to be a little bit more active, whereas before [diagnosis] I might have thought ‘I won’t do any exercise tonight’, I think ‘right I’ve got to do it because of the bronchiectasis’.”

[B10]

Another critical motivator was being healthy for family reasons:

“The idea, certainly with the grandchildren, is to be as fit as you can around them so that you can keep up with them! It has been quite physically demanding [looking after the grandchildren].”

[B8]

In line with the bronchiectasis participants, healthy adults also highlighted an understanding of the importance of exercise:

“The benefit [of exercising] is I know it is good for me. It’s probably the best drug in the world, if you could bottle it. I do it for my own health and that it makes me feel better about myself.”

[H5]

##### External Influences

External influences to exercise also centred around health, implications of ageing and the impact a person’s health can have on others:

“I think there is a moral obligation to exercise and at least remain healthy, because it matters to other people what happens to us. Obesity and smoking causes demand on the health service… problems just get worse as you get older and then it drags other people in because they have to look after you… It’s a drag on your friends and relatives and you have to consider the effect of your own decisions on other people.”

[H9]

Furthermore, the social aspect and the influences of others were also highlighted as an external motivator to exercise:

“It depends who you hang around with. If you hang around with lots of sports people you tend to learn off your group. Whereas, if you haven’t got that social side to it, then maybe you won’t exercise as much.”

[H1]

Some healthy participants also discussed the pressures they feel to be active, citing external influences such as media, society pressures and weight control:

“If I wasn’t particularly active, I think I would feel guilty. I don’t know if that is something that is put on me myself or more because of the media aspect… I’m probably going to have more health complications, probably going to be overweight. I don’t know if it is a societal thing or a personal thing [pressure to be active], but I’d feel guilty if I didn’t [exercise].”

[H1]

#### 3.3.2. Inspiratory Muscle Training Intervention

##### Enjoyment

Bronchiectasis participants expressed ‘enjoyment’ of undertaking IMT and that it was something they ‘looked forward’ to due to the perception it could help their health:

“I was looking forward to doing it, because I want to do anything that’s going to help. I’ve been so poorly with my chest this year that anything that will help, I will try.”

[B2]

##### Motivations

Bronchiectasis patients discussed determination and motivation to take part in the training, due to the structure of the training programme:

“I was determined, I wanted to do this [IMT]… the main reason is that it gives me that discipline, I am more than happy to follow a training programme and then the motivation followed ‘I will do this’.”

[B3]

Participants enjoyed the high-intensity nature of the training and discussed how the differing levels and reducing rest times were ‘challenging’ but motivated them to try harder:

“They [decreasing rest times] were good. That really set a goal for you, you know. It was hard, but I was motivated to do it, it did push you a bit more!”

[B1]

“That made it harder at the time [levels and decreasing rest times]. I think it was good, it kept it more engaging… it kind of has a reward element to it, I suppose.”

[H9]

In contrast to bronchiectasis, while some enjoyed the IMT intervention, more healthy participants discussed having less motivation to complete training compared to regular exercise, due to the lack of intrinsic and extrinsic rewards:

“Less motivated to do it [IMT] than I am to do exercise. I think once you’ve done exercise you feel good about it afterwards, you feel like you have achieved something. Whereas, after doing the PrO2 training, it just felt like something that I had to do, I had to get through, but no reward at the end.”

[H1]

The reasons for this lack of motivation were due to participant’s perceived lack of improvements from undertaking IMT, with healthy participants suggesting that clinical populations or athletes may benefit from this style of training more:

“I’m guessing you might get more significant results if someone sort of sedentary was using it. I don’t feel like it was helping me in anything other than being able to do the test better.”

[H6]

##### Accessibility

The remote nature of the training programme was a positive for the participants as it allowed easy integration into their daily lives, with participants reporting that if it was not home-based, they would not have taken part:

“If I had to go somewhere to use it, I probably would not have done it… I would have had to have made more of an effort and having something small and portable, why would I want to go somewhere else with it?... Having it at home I can do it easily”

[B4]

Similarly, the convenience of being able to train at home was also discussed by healthy participants as a positive of the intervention:

“I think it’s better to have it at home than having it elsewhere, it would never have worked [training elsewhere]… It’s just convenient really, doing it at your own time at your own pace without anyone watching.”

[H2]

Healthy participants also discussed the ease of training and how easily it can be integrated into daily routine:

“It’s quite portable and convenient as opposed to setting an hour of your day aside to physically go and do exercise… It’s a lot more flexible in terms of time of day or motivation. You can adapt to it quite well.”

[H10]

##### Feedback

Another highlight of the training was the accompanying app and the live feedback loop it provided. All participants cited this as a critical motivating factor, while giving them a chance to set their own goals:

“You can set your goals then… I found having that sort of feedback was very helpful”

[B7]

“It [feedback] changed my behaviour for the better, I was aiming for something, I knew what I was meant to be doing. I think having that goal in mind helps the person try and exercise.”

[H7]

While it gave participants an opportunity to track their own progress, it also brought out the competitive nature of some participants:

“It is nice to know how you are doing, but it’s also nice to have something to compete against. If somebody would have asked me before ‘are you competitive’? I would have said no, not really, but yes, in this instance I certainly am!”

[B5]

Participants also highlighted that if they did not have this extrinsic motivation, they would not have trained or have been as motivated to try as hard as they perceived they did:

“You wouldn’t have pushed yourself as much [if you didn’t have the visual feedback]”

[B4]

“I wouldn’t have tried as hard because you don’t know how you’ve done. You wouldn’t be as motivated to try then.”

[H2]

However, healthy participants also discussed the negative aspects of the feedback element and how it could also potentially be demotivating:

“Then I got really frustrated if I couldn’t get to the end.”

[H4]

#### 3.3.3. Perceived Improvements

Importantly, all bronchiectasis participants perceived an improvement in their physical ability and health after completing IMT, attributing this solely to the training programme:

“I’m not doing anything different in my life other than using the device… Some of the things I do now, the recovery time after doing them is about half [the recovery time] was before. I have never got this far into a winter without a cold turning into a chest infection. My lungs aren’t producing the same levels of mucus as they typically do which is a great improvement. Physically I’m feeling better, now that’s [IMT] got to be worth doing.”

[B8]

Activities of daily living were also perceived to be easier:

“My stamina has got better I would say that, I was quite pleased with that actually. I found it easier walking up the stairs, I was fitter. It’s all hills round me and two months ago I wouldn’t have been able to do an hour [walk]… but now it’s ok!”

[B1]

Bronchiectasis participants also voiced the ability to expectorate sputum more easily, which they felt led to a decrease in recurrent respiratory infections:

“What I like about it was that it helped me to clear a lot of sputum much better than the Acapella [an airway clearance device]… I haven’t had any infections, and this is my tenth week without antibiotics or infection. I think my record before was three weeks!... When you have bronchiectasis, as long as you can keep your lungs clear, happy days. That is the most important thing.”

[B2]

Some healthy participants (38%) also discussed perceived improvements post-IMT:

“I definitely think my lung capacity is improved… I decided to go for a little run and I was really shocked my breathing had improved… I definitely felt the benefit from it, which was surprising actually. I think it has definitely made a difference. I know a couple of friends of mine are quite jealous.”

[H10]

However, they could not be confident that their perceived improvements were attributable to IMT alone:

“There’s definitely been an improvement in my training over time, but I don’t know whether that was necessarily because of this device.”

[H5]

#### 3.3.4. Future Improvements

##### Adherence

Despite the positives, participants did voice some areas they would like to improve. Specifically, some bronchiectasis patients, despite excellent adherence to the training programme, thought the training programme was ‘too long’ (30%):

“Tiresome three times a week… it was getting to be a bit of a pain after eight weeks… Possibly if it could get condensed to fit into your lifestyle more.”

[B4]

Which was also voiced by the healthy participants and would have potentially helped with adherence in this cohort (75%):

“If you could condense it into a shorter training period, that would definitely help in making me complete more sessions.”

[H5]

##### Extrinsic Feedback

Participants, irrespective of health status, suggested more extrinsic feedback to help with motivation, while also providing rewards:

“I would have liked something like a smiley face or a ‘hip hip hooray! You’ve done it, well done’!”

[B1]

“I think from a sort of motivation point of view, it may sound silly, but just to have a ‘well done, that was better than last time, you’ve improved this much,’ ‘you’re nearly there, keep on going,’ and ‘you’re doing better than you have previously done.’ So, that would help motivate you.”

[H6]

Despite participants exhibiting goal setting, they would also like the app to provide tangible goals that they have to reach:

“If it set you targets to achieve within a certain period. That would make it far more worthwhile.”

[B4]

“You could have a set target from the data… you know ‘can I get closer to the target’? Without targets, it was difficult to know what you could improve on or whether you were improving.”

[H9]

## 4. Discussion

This study revealed that an eight-week, home-based IMT programme elicited significant and clinically meaningful increases in inspiratory muscle strength and endurance in both bronchiectasis and healthy participants, with healthy participants also significantly enhancing expiratory flow and exercise capacity. Whilst no additional adaptations were shown at 16 weeks, IMT once per week was sufficient to maintain those benefits already elicited. These physiological benefits were enabled by a high adherence to the IMT protocol, with participants reporting that IMT was enjoyable and that they perceived health and well-being benefits. This therefore suggests that IMT may represent an effective and palatable alternative to traditional pulmonary rehabilitation programmes in those with bronchiectasis.

As bronchiectasis is associated with respiratory muscle weakness [5,6,7], the clinically meaningful improvement in MIP observed after eight weeks of IMT in the current study is of clinical importance. A recent systematic review and meta-analysis concluded that RMT may improve the strength of the respiratory muscles [16], specifically low-intensity IMT (30% MIP) improved MIP by 39% [5], while a progressive overload-based IMT programme (20–70% MIP) elicited improvements of 44% [6]. However, in contrast to the present study, both Newall (2005) [5] and Ozalp et al. (2019) [6] found significant improvements in MEP of 44% and 12%, respectively. Indeed, whilst the present study found improvements in MEP, these were not statistically significant or clinically meaningful. Such discrepancies may be due to the small sample size in the present study, though it is noteworthy that these findings are in accord with Harver and colleagues (1989) [36] who found that eight weeks of high-intensity IMT improved MIP, but not MEP, in individuals with COPD. As such, the physiological effect of IMT on expiratory muscles remains unclear. It has, however, been hypothesised that inspiring against a resistance, as is the case in IMT, could increase the activation of the expiratory muscles by force extension, leading to an increase in MEP [6]. Elsewhere, it has been postulated that increased inspiratory muscle strength results in better thorax expansion, enabling a greater elastic recoil, that could improve MEP and PEF [37]. Decreased expiratory muscle strength can have detrimental effects in bronchiectasis patients on their effectiveness of coughing and removal of airway secretions [8,9], therefore, further research is required to determine the effect, and underlying mechanism, IMT has on respiratory muscle function.

Lung function parameters were not affected by IMT in the present intervention. This is in line with previous research that found eight weeks of high-intensity IMT increased MIP but had no effect on FEV_1_ or FVC in those with COPD [36], CF [37] or bronchiectasis [7]. Despite there being no significant improvements in respiratory function post-IMT, MIP values were significantly associated with pulmonary function in those with bronchiectasis. It could therefore be postulated that those with inspiratory muscle weakness may suffer from more severe airflow obstruction and greater lung hyperinflation. This is in accord with previous research that found a positive correlation between MIP and airflow limitation in a COPD population [38,39].

Both SMIP and inspiratory duration are emerging as key clinical markers across several populations [19,40], with suggestions that they are both superior markers of inspiratory performance than MIP [41]. In combination, they provide a more comprehensive assessment of identifiable characteristics of respiratory muscle weakness or fatiguability [40], while potentially providing greater ability to identify outcomes associated with mortality risk in individuals with COPD [41,42]. The significant improvements in SMIP and inspiratory duration exhibited post-IMT in the present study are therefore of particular interest. In COPD, greater SMIP is independently related to reduced airflow limitation, less dyspnoea and increased six-minute walk test (6MWT) distance [42]. Like SMIP, increased inspiratory duration is reported to be associated with an increase in distance walked in 6MWT [42]. Therefore, the current improvements could be postulated to translate to meaningful enhancements in functional exercise capacity.

Exercise intolerance is often reported in those with bronchiectasis, the basis for which is likely to be multifaceted and includes decreased ventilatory efficiency, altered respiratory mechanics, insufficient gas exchange, expiratory flow limitation and increased dynamic hyperinflation [2,4]. Earlier studies have reported significant improvements in intermittent shuttle walk test distance in people with bronchiectasis [5,6]. However, congruent to earlier studies [7], IMT was not associated with any change in VO_2max_ in those with bronchiectasis in the current study, despite participants reporting perceived improvement in their physical ability to undertake activities of daily living. These apparent discrepancies in the effect of IMT on exercise capacity are perhaps more likely to be attributable to the measure of exercise capacity utilised within the studies. Specifically, the current cardiopulmonary exercise testing (CPET)-derived values provide an accurate measure of maximal exercise capacity, the clinical significance of which is increasingly evident with regards to predicting prognosis in numerous clinical conditions [43]. However, the applicability of this measure to functional capacity and daily activities has been questioned [44], with suggestions that measures such as the 6MWT distance may be more applicable to bronchiectasis due to its relatedness to everyday activity [45]. The discrepancies in the effect of VO_2max_ are contradictory to what might be expected, given the well-established relationship between baseline fitness and the magnitude of change elicited by traditional exercise interventions [46]. Therefore, it could be speculated that those with bronchiectasis could have different sites or mechanisms of exercise intolerance, that are perhaps less effected by IMT or require a greater dose, be that in terms of intensity, frequency, or duration, for beneficial changes to be manifested. Therefore, future IMT studies should consider incorporating measures of functional capacity and assessing the effect of IMT on the underlying causes of functional capacity limitations.

Although the value of patient involvement in clinical research is well established [47], few studies have specifically explored the opinions, experiences and/or needs of those with bronchiectasis [48]. Moreover, whilst Hoffman et al. (2018) [10] found that individuals with chronic lung disease perceived IMT to be of benefit and to increase their confidence in their disease management, our understanding of patients’ motivations to complete IMT is limited. More specifically, no research has previously applied or examined motivation theories within this population. Importantly, IMT elicited high levels of intrinsic motivation in the bronchiectasis participants in this study, who endorsed and valued their involvement in the intervention, reporting satisfaction, interest and engagement. A critical motivating factor for participants desire to train was the live biofeedback, allowing the adoption of mastery orientated goals, which have been shown to be positively associated with effort in physical activity and exercise interventions [49]. Furthermore, high levels of motivation were reflected in the increased levels of autonomy and perceived competence and low levels of amotivation and controlled regulation in bronchiectasis compared to healthy participants. High levels of autonomy have been associated with positive health, behavioural and psychological outcomes, such as adherence to treatment regimens [50]. Contrastingly, controlled forms of amotivation, as reported in the healthy group, have been linked to poorer adherence [51]. The differences in motivation between groups could explain the better adherence noted in bronchiectasis participants. Indeed, enjoyment, enhanced autonomy and competency are important factors for long-term adherence in clinical populations [52,53]. Such high adherence to the IMT protocol in those with bronchiectasis is discordant to research utilising other treatment regimes in this population [54]. Adherence to such treatment programmes has also been associated with pulmonary exacerbations [54], reinforcing the potential utility of IMT as a viable rehabilitation strategy for those with bronchiectasis.

Previous research found that patients with bronchiectasis experience feelings of anxiety and fear of exacerbation [55]. Indeed, the main motivations for being physically active and to undertake IMT in our cohort were centred around the management of their bronchiectasis and their desire to improve their health. Patients reported perceived improvements in their physical ability, a better ability to expectorate sputum in comparison to using traditional methods, such as the Acapella^®^ (Smiths Medical, Wampsville, NY, USA), and a reduction in exacerbations during the intervention, which they attributed solely to IMT. Of importance, impaired clearance of sputum results in a vicious cycle of colonisation and infection of the bronchi with pathogenic organisms, dilation of bronchi, pulmonary exacerbations and further production of sputum [56] and therefore the ability to remove sputum is a key finding. Indeed, this is congruent with previous research which found that training protocols incorporating ≥80% MIP are better at enhancing mucus transport, due to higher flow rate as a result of the higher pressure elicited [57]. Conversely, previous research found that sputum clearance only increased with the Acapella^®^, which was preferred by patients compared to threshold IMT [57]. However, it is pertinent to note that sputum clearance was self-reported in the present study, which is subject to bias. Future IMT research should therefore utilise sputum volume measurements.

Reductions in reported exacerbations as a result of the IMT intervention is also an important finding. Indeed, the economic burden of exacerbation in those with bronchiectasis resulting in hospitalisations is significant [58]. Unsurprisingly, the convenience of being able to train at home was highlighted as a key enabler, particularly in a population with increasing treatment burdens [59]. Therefore, the cost-effective, home-based nature of IMT could be advantageous in those with bronchiectasis.

Despite numerous strengths, this study is not without its limitations. The lack of age- and sex-matched controls could explain the discrepancies in exercise capacity and, more specifically, the lack of perceived physical improvement in the healthy cohort. Indeed, the significantly younger healthy population mean such results should be interpreted with caution. Despite the recruitment of age- and sex-matched controls, the onset of COVID-19 precluded the completion of post-intervention testing. It should also be noted that an a-priori power calculation suggested that a sample size of 13 participants per experimental group was required. Further, the implementation of an IMT protocol is likely to be associated with a learning effect. However, the magnitude of change observed in the current study exceeds those anticipated, suggesting changes are unlikely to be solely attributable to learning effects. Finally, the relatively small sample size limits statistical power and interpretation of the data. However, the application of MCIDs enables studies to be adequately powered with fewer participants [60,61] and has been highlighted as more important for future treatment decisions [62].

## 5. Conclusions

In conclusion, an eight-week IMT programme significantly increased inspiratory strength, respiratory endurance, perceived competence and autonomy in those with bronchiectasis. Furthermore, bronchiectasis patients perceived an improvement in their physical ability and had high levels of intrinsic and extrinsic motivation to complete IMT. IMT, therefore, appears to be an effective and palatable tool to enhance physiological and psychological health in those with bronchiectasis.

## Figures and Tables

**Table 1:** Baseline anthropometrics of adults with bronchiectasis and healthy participants.

Characteristics	Total (*n* = 18)	Bronchiectasis (*n* = 10)	Healthy (*n* = 8)	*p* Value
**Anthropometrics**				
Age (years)	51.9 ± 17.2	64.5 ± 10.3	36.1 ± 8.5	<0.05 *
Sex (female/male)	(5/13)	(3/7)	(2/6)	0.886
Height (cm)	172.6 ± 10.9	169.5 ± 11.2	176.4 ± 9.7	0.183
Weight (kg)	76.9 ± 16.8	81.2 ± 19.7	71.5 ± 11.2	0.238
BMI (kg·m^−2^)	25.8 ± 5.5	28.3 ± 6.3	22.8 ± 1.8	0.024 *
WHR (cm)	1.0 ± 0.1	1.0 ± 0.1	0.9 ± 0.6	0.106

BMI, body mass index; WHR, waist-to-hip ratio. * *p* < 0.05 (significant difference between Bronchiectasis and Healthy groups).

**Table 2:** Meaningful clinical difference between baseline, eight weeks and 16 weeks in adults with bronchiectasis.

Parameters	Mean Difference from Baseline–8 Weeks (*n* = 10)	% of Participants with Clinically Significant Increase	% of Participants with Clinically Significant Decrease	Effect Size	Mean Difference from 8 Weeks–16 Weeks (*n* = 3)	% of Participants with Clinically Significant Increase	% of Participants with Clinically Significant Decrease	Effect Size
**Lung Function**								
MIP (cmH_2_O)	19.6 ± 18.9 *	40%	0%	0.4	23.7 ± 27.5	0%	33%	0.5
MEP (cmH_2_O)	3.2 ± 20.3	20%	10%	0.1	4.7 ± 4.1	0%	0%	0.2
SMIP (PTU)	66.1 ± 57.8 *	50%	0%	0.7	65.8 ± 57.6	0%	0%	0.2
ID (s)	3.8 ± 4.0 *	60%	0%	0.2	3.4 ± 4.1	0%	0%	0.1
FEV_1_ (l)	0.01 ± 0.2	0%	0%	0.01	0.1 ± 0.1	0%	0%	−0.1
FEV_1_ %predicted	0.4 ± 11.7	30%	60%	0.02	7.0 ± 6.7	67%	0%	0.5
FVC (l)	0.01 ± 0.2	10%	0%	−0.02	0.1 ± 0.1	0%	0%	−0.2
FVC %predicted	−2.0 ± 14.1	40%	40%	−0.2	9.0 ± 13.1	33%	0%	0.9
PEF (L·min^−1^)	5.0 ± 49.7	20%	10%	0.04	5.0 ± 9.5	0%	0%	−0.04
FEV_1_/FVC	2.1 ± 10.2	10%	0%	0.1	1.3 ± 1.5	0%	0%	0.1
**Exercise Capacity**								
Vo_2_ max (ml·kg^−1^·min^−1^)	0.1 ± 1.7	10%	10%	−0.04	4.3 ± 5.1	0%	100%	2.0

MIP, maximal inspiratory pressure; MEP, maximal expiratory pressure; SMIP, sustained maximal inspiratory pressure; PTU, pressure time unit; ID, inspiratory duration; s, seconds; FEV_1_, forced expiratory volume in one second; FVC, forced vital capacity; PEF, peak expiratory flow; Vo_2max,_ maximal oxygen uptake. * *p* < 0.05.

**Table 3:** Self-determination measures after eight weeks of inspiratory muscle training.

	8 Weeks				95% Confidence Interval		*p* Value between Groups
	**Bronchiectasis**	**Healthy**	**Mean Difference**	**SEM**	**Lower**	**Upper**	
Treatment Self-Regulation							
Autonomous	4.9 ± 1.4 *	2.1 ± 0.7	2.8	0.5	1.7	4.0	<0.01 *
Controlled	1.7 ± 0.7	1.5 ± 0.3	0.2	0.3	−0.4	0.8	0.411
Amotivation	1.4 ± 0.6	4.3 ± 0.4 *	−2.9	0.3	−3.4	−2.4	<0.01 *
Perceived Competence	5.5 ± 1.3 *	4.6 ± 1.1	0.9	0.6	−0.31	2.1	0.128

SEM, standard error of the mean. * *p* < 0.05 within group differences.

## Data Availability

The data presented in this study are available on request from the corresponding author. The data are not publicly available due to anonymity and confidentiality considerations.

## Supplementary Materials

The following are available online at https://www.mdpi.com/1660-4601/18/6/3051/s1, Table S1: Effect of an eight-week inspiratory muscle training intervention in adults with bronchiectasis and healthy participants.
