# Supplementary material for: The Effect of a High-Intensity PrO2Fit Inspiratory Muscle Training Intervention on Physiological and Psychological Health in Adults with Bronchiectasis: A Mixed-Methods Study
Source: Int J Environ Res Public Health. 2021 Mar 16;18(6):3051. doi: 10.3390/ijerph18063051 (PMC8001489; doi:10.3390/ijerph18063051)
Supplement: Supplementary file 1 [file ijerph-18-03051-s001.pdf]

Table S1. Effect of an eight-week inspiratory muscle training intervention in adults with bronchiectasis and healthy participants.

Parameters	Baseline		8-Week		Change from Baseline Mean	
	Bronchiectasis (n = 10)	Healthy (n = 8)	Bronchiectasis (n = 10)	Healthy (n = 8)	Bronchiectasis (n = 10)	Healthy (n = 8)
MIP (cmH_2O)	72.5 ± 44.5	95.3 ± 46.8	92.1 ± 50.8	125.9 ± 35.2	19.6 ± 18.9*	30.6 ± 29.3 *
MEP (cmH_2O)	65.7 ± 22.3	107.5 ± 56.8	68.9 ± 25.9	115.6 ± 49.5	3.2 ± 20.3	8.1 ± 28.3
SMIP (PTU)	407.2 ± 354.2	437.0 ± 206.0	473.4 ± 327.0	513.2 ± 238.0	66.1 ± 57.8 *	776.2 ± 80.8 *
Inspiratory Duration (s)	10.4 ± 5.7	12.2 ± 2.7	14.2 ± 5.3	15.9 ± 4.6	3.8 ± 4.0*	3.8 ± 2.7*
FEV_1 (l)	2.5 ± 0.6	3.8 ± 0.9	2.5 ± 0.6	3.8 ± 1.0	−0.01 ± 0.2	0.01 ± 0.3
FEV_1 %predicted	78.1 ± 15.8	90.6 ± 8.9	78.5 ± 15.5	90.9 ± 15.8	0.4 ± 11.7	0.3 ± 8.3
FVC (l)	3.2 ± 0.6	4.8 ± 1.2	3.2 ± 0.7	4.8 ± 1.2	−0.01 ± 0.2	0.02 ± 0.5
FVC %predicted	80.3 ± 11.9	93.8 ± 12.5	78.3 ± 10.4	94.8 ± 9.1	−2.0 ± 14.1	1.0 ± 7.8
PEF ($L \cdot min^{-1}$)	436.5 ± 127.3	547.4 ± 107.9	441.5 ± 105.0	557.6 ± 110.3	5.0 ± 49.7	10.3 ± 4.8 *
FEV_1/FVC	80.3 ± 15.4	82.7 ± 15.3	82.4 ± 14.8	79.3 ± 15.8	2.1 ± 10.2	−3.4 ± 10.6
$\dot{V}O_{2max}$ ($ml \cdot kg^{-1} \cdot min^{-1}$)	11.1 ± 2.6	39.7 ± 11.0	11.0 ± 2.2	44.3 ± 13.6	−0.1 ± 1.5	4.6 ± 4.2 * #

MIP, mean inspiratory pressure; MEP, mean expiratory pressure; SMIP, sustained maximal inspiratory pressure; PTU, pressure time unit; ID, inspiratory duration; s, seconds; FEV_1, forced expiratory volume in one second; FVC, forced vital capacity; PEF, peak expiratory flow; $\dot{V}O_{2max,}$ maximal oxygen uptake. * $p < 0.05$ change from baseline; # $p < 0.05$ for change between groups.
